# PRDM Proteins: Molecular Mechanisms in Signal Transduction and Transcriptional Regulation

**DOI:** 10.3390/biology2010107

**Published:** 2013-01-14

**Authors:** Erika Di Zazzo, Caterina De Rosa, Ciro Abbondanza, Bruno Moncharmont

**Affiliations:** 1 Department of Medicine and health sciences, University of Molise, via De Sanctis snc, Campobasso 86100, Italy; E-Mail: erika.dizazzo@unimol.it; 2 Department of Biochemistry, Biophysics and general Pathology, Second University of Naples, via L. De Crecchio 7, Napoli 80138, Italy; E-Mails: caterina.derosa@unina2.it (C.D.R.); ciro.abbondanza@unina2.it (C.A.)

**Keywords:** PRDM gene family, signal transduction, transcriptional regulation

## Abstract

PRDM (PRDI-BF1 and RIZ homology domain containing) protein family members are characterized by the presence of a PR domain and a variable number of Zn-finger repeats. Experimental evidence has shown that the PRDM proteins play an important role in gene expression regulation, modifying the chromatin structure either directly, through the intrinsic methyltransferase activity, or indirectly through the recruitment of chromatin remodeling complexes. PRDM proteins have a dual action: they mediate the effect induced by different cell signals like steroid hormones and control the expression of growth factors. PRDM proteins therefore have a pivotal role in the transduction of signals that control cell proliferation and differentiation and consequently neoplastic transformation. In this review, we describe pathways in which PRDM proteins are involved and the molecular mechanism of their transcriptional regulation.

## 1. Structure of PRDM Proteins and Their Alternative Gene Products

The PRDM (PRDI-BF1 and RIZ homology domain containing) protein family is characterized by the presence of an *N*-terminal PR (PRDI-BF1 and RIZ1 homology) domain. The PR domain shares high homology with the catalytic SET (Suppressor of variegation 3–9, Enhancer of zeste and Trithorax) domain that defines a group of histone methyltransferases [1]. In the human genome there are 17 genes encoding for proteins with a PR/SET and all of them but PRDM11 have a variable number of Zn-finger domains [2]. PRDM proteins have a pivotal role in the transduction of signals that control cell proliferation and differentiation and consequently neoplastic transformation [3]. A common characteristic of *PRDM* family genes is the expression of different molecular forms by alternative splicing or by the action of different promoters. Furthermore, some genes of this family are expressed as two alternative forms, one lacking the PR domain (PR-*minus*) but otherwise identical to the other PR-containing product (PR-*plus*) (*PRDM1*, *PRDM2*, *PRDM3*, *PRDM16*) [4,5,6,7]. Others genes encode for proteins that differ for the presence or absence of Zn-finger domains (*PRDM6*, *PRDM9*) [8,9]. Recent reviews presented schematic diagrams showing the main PRDM gene products [2,3,10].

### 1.1. Alternative Promoters

*PRDM1* and *PRDM2*, initially identified as Blimp-1 (B lymphocyte-induced maturation protein-1) and RIZ (Retinoblastoma interacting zinc finger protein) respectively, have two promoters that encode for a PR-*plus* and a PR-*minus* isoform. *PRDM1* promoters are localized upstream of exon 1 and exon 4 respectively. These transcriptional start sites at two promoters guide: PRDI-BF1 (Positive regulatory domain I-binding factor 1) α (PR-*plus*) e PRDI-BF1β (PR-*minus*) that differ only by the PR domain presence [4,11]. One promoter of *PRDM2* is located upstream of the open reading frame in a region including exon 1a and a second promoter is located within intron 5 and exon 6 [6]. Similarly to *PRDM1*, *PRDM2* expresses two proteins, PRDM2a/RIZ1 (PR-*plus*) and PRDM2b/RIZ2 (PR-*minus*), by differential transcription initiated by the two promoters.

*PRDM16* encodes a Zn-finger protein (MEL1) that shares 63% sequence similarity to *PRDM3/MECOM* (MDS1 and EVI1 complex locus, also known as *EVI1*, *MDS1*). Like *PRDM3*, two mRNAs coding for PR-*plus* and PR-*minus* protein are transcribed from this locus: PRDM16/MEL1 (MDS1/EVI1-like gene 1), the PR-*plus* form, with the PR domain coded from codon ATC91 (exon 2) to codon CCC223 (exon 5) and PRDM16/MEL1S, the PR-*minus* form, initiated from an internal codon ATG599 (exon 9) [12,13].

### 1.2. Alternative Splicing

*PRDM1* encodes also for an alternatively spliced transcript lacking exon 7; this variant (Blimp-1Δexon7) lacks DNA binding activity and fails to bind G9a or HDAC1/2, but retains the ability to interact with Prmt5 (protein methyltransferase 5) [14]. This evidence suggests that the expression of *PRDM1* alternative splicing variants is regulated during development by chromatin structure modification and fine-tunes *PRDM1*’s functional capabilities [14].

*PRDM3/MECOM* is a complex locus containing *EV1* and *MDS1* genes, located on chromosome 3q26. This complex locus encodes for different gene products generated by alternative splicing or by intragenic splicing [15].

The major and most studied protein, EVI1 (Ecotropic virus integration site 1 protein homolog), also named MECOM (E) is a 1051 aminoacid protein [16], that consists of an *N*-terminal seven-zinc finger domain, a central transcription repression domain, a second zinc finger domain with three finger motifs and a *C*-terminal acidic region. One EVI1 mRNA splice variant, identified as PRDM3/EVI1/Δ324, is a protein that lacks zinc fingers 6 and 7 as well as part of the transcription repression domain. The PRDM3/EVI1-Rp9 variant is abundant both in humans and mice and lacks 9 amino acids in the repression domain. The variant, PRDM3/EVI1/Δ105, is a protein truncated of 105 aminoacids at its *C*-terminus and is detected only in murine but not in human cells.

*EVI1* may form a fusion transcript with the *MDS1* gene located upstream. The use of alternative transcriptional start sites generates mRNA combining sequences derived from the *MDS1* (*Myelodysplasia syndrome-associated protein 1*) gene, which is located upstream of *EVI1*, and the EVI1 sequences starting from exon 2. The derived protein, called MDS1/EVI1 or MECOM (ME), from this mRNA contains a 188 amino acid extension encoding a PR domain at its *N*-terminus, but is otherwise identical to the EVI1 protein [5,15,17].

In mice, *Prdm6* encodes for four isoforms referred to as Prdm6/4#, 3#, 33# and 36#, produced by alternative splicing. Prdm6/4# has a PR/SET domain in the central region and four Zn-finger domains at its *C*-terminal region. Prdm6/3# and Prdm6/33# have an additional sequence of 31 residues produced by retention of the first intron, absent in the Prdm6/4# transcript. Similarly, Prdm6/36# has a single amino acid insertion if compared to Prdm6/4#, derived from the recognition of an alternative splicing site 3 bp upstream of the intron 1/exon 2 boundary. Prdm6/33# is a PR-*minus* isoform, obtained by an alternative splicing event that results in the deletion of exons 3–5 of transcript encoding for Prdm6/4#. Prdm6/36#, missing the fourth Zn-finger domain, derives from an alternative splicing in intron 7 of Prdm6/4# transcript that includes an in-frame stop codon [9].

The *Prdm9* murine gene encodes for three isoforms generated by alternative splicing: one isoform has a PR domain in its *N*-terminal region and a Zn-finger motif in its *C*-terminal portion. The other two isoforms generated by alternative splicing lack the Zn-finger domain responsible of the nuclear localization [8].

In 2002 Siegel, analyzing the protein extracted from mouse brain by Western blot with an antibody to the *C*-terminal region of Prdm10/tristanin, identified two molecular forms of this protein of 50 kDa and 25 kDa respectively. This finding corroborates the hypothesis that there are also different molecular variants encoded by the gene *Prdm10* [18].

The functional relevance of the different variants has not yet been elucidated. Table 1 summarizes the information relative to PRDM proteins obtained from Uniprot [19] and National Center for Biotechnology Information protein database [20].

**Table 1:** PRDM (PRDI-BF1 and RIZ homology domain containing) proteins derived by alternative promoters activity or alternative splicing.

Human								
*gene name*	*protein name*	*localization*	*molecular forms*	*alternative promoter (UNIPROT entry)*	*splicing variants (UNIPROT entry)*	*length (aa)*	*PR domain*	*HMT activity*
*PRDM1* (*BLIMP1*)	PR domain zinc finger protein 1 (*BLIMP1*)	nucleuscytoplasm	3	Isoform 1 'canonical' sequence (O75626-1)	Isoform 2	825	aa 85-205	no
					1-36: missing			
	(Beta-interferon gene positive regulatory domain I-binding factor)				(O75626-2)			
					Isoform 3		partially missing	no
					1-3: MLD → MEK			
	(PR domain-containing protein 1)				4-137: missing			
	Positive regulatory domain I-binding factor 1)				(O75626-3)			
*PRDM2* (*KMT8*, *RIZ*)	PR domain zinc finger protein 2	nucleus	3	Isoform 1 (RIZ1) 'canonical' sequence (Q13029-1)	Isoform 2 (MTB-Zf)	1,718	aa 27-145	H3K9
	(GATA-3-binding protein G3B)				1679-1682: SYSL → RNFL			
					1683-1718: missing *			
	(Lysine *N* - methyltransferase 8)				(Q13029-2)			
	(MTB-ZF)			Isoform 3 (RIZ2) 1-201: missing (13029-3)			no	no
	(MTE-binding protein)							
	(PR domain-containing protein 2)							
	(RIZ, Retinoblastoma protein-interacting zinc finger protein)							
*PRDM3*/*MECOM* (*EVI1*)	MDS1 and EVI1 complex locus protein EVI1 (Ecotropic virus integration site 1 protein homolog-EVI-1)	nucleus	6		Isoform 1 (Evi-1a) 'canonical' sequence	1,051	aa 79-194	H3K9me1
					(Q03112-1)			
					Isoform 2 (Evi-1c) (Mds1/Evi1)			
					(Q03112-3)			
					1-1: M → MRSKGRARKL...APGEELLL-FM			
					Contains an additional SET domain at positions 79-194			
					Isoform 3 (Mds1)			
					(Q13465-1)			
					Isoform 4			
					1-1: M → MILDEFYNVKFCIDASQPD-VGSWLKYIRFAGCYDQHNLVACQINDQIFYRVVADIAPGEELLLFM			
					138-138: K → KQ			
					(Q03112-4)			
					Isoform 5			
					672-680: missing			
					(Q03112-5)			
					Isoform 6			
					138-138: K → KQ			
					672-680: missing			
					(Q03112-6)			
*PRDM4* (*PFM1*)	PR domain zinc finger protein 4 (PR domain-containing protein 4)	nucleus	1			801	aa 412-533	no
*PRDM5* (*PFM2*)	PR domain zinc finger protein 5 (PR domain-containing protein 5)	nucleus	3		Isoform 1 'canonical' sequence	630	aa 8-128	no
					(Q9NQX1-1)			
					Isoform 2			
					218-248: missing			
					(Q9NQX1-2)			
					Isoform 3 (Q9NQX1-3)			
					101-111: EGENIFYLAVE → DKNLGP-AEWRG			
					112-630: missing			
*PRDM6* (*PFM3*)	Putative histone-lysine *N* -methyltransferase PRDM6	nucleus	3		Isoform 1 (Q9NQX0-3)	595	aa 247-369	H4K20
					'canonical' sequence			
	(PR domain zinc finger protein 6)				Isoform 2 (B)			
					1-182: missing			
	(PR domain-containing protein 6)							
					314-595: missing			
					(Q9NQX0-2)			
					Isoform 3 (A)			
					1-182: missing			
					(Q9NQX0-1)			
*PRDM7* (*PFM4*)	Probable histone-lysine *N* - methyltransferase PRDM7 (PR domain zinc finger protein 7) (PR domain-containing protein 7)	nucleus	3		Isoform 1 'canonical' sequence	492	aa 246-362	no
					(Q9NQW5-3)			
					Isoform 2 (B)			
					1-206: missing			
					318-377: YVNCARDDEE...RSSIEPAESL → TKARDPSMSL...RGSESGaaIF			
					378-492: missing			
					(Q9NQW5-2)			
					Isoform 3 (A)			
					1-206: missing			
					368-492: RSSIEPAESL...VKRSKKGPNS → KWGSKWKKEL...GEAPVCRKDE			
					(Q9NQW5-1)			
*PRDM8* (*PFM5*)	PR domain zinc finger protein 8	nucleus	2		Isoform 1 'canonical' sequence	aa 8-135	H3K9	
					(Q9NQV8-1)			
					Isoform 2			
	(PR domain-containing protein 8)				332-334: GRG → aaL			
					335-689: missing			
					(Q9NQV8-2)			
*PRDM9* ( *PFM6* )	Histone-lysine *N* -methyltransferase PRDM9	nucleus	1		(Q9NQV7)	894	aa 246-362	H3K4me3
	(PR domain zinc finger protein 9;							
	PR domain-containing protein 9)							
*PRDM10* (*KIaa 1231*; *PFM7*; *TRIS*)	PR domain zinc finger protein 10 (PR domain-containing protein 10) (Tristanin)	nucleus	6		Isoform 3 'canonical' sequence	1,147	aa 206-330	no
					(Q9NQV6-3)			
					Isoform 2			
					1-97: MDSKDESSHV...AYVQQDATAQ → MSAYSVPSTFA			
					511-514: missing			
					952-985: missing			
					(Q9NQV6-2			
					Isoform 1			
					1-97: MDSKDESSHV...AYVQQDATAQ → MSAYSVPSTFA			
					(Q9NQV6-1)			
					Isoform 4			
					511-514: missing			
					984-984: I → IQVSEPTASAPSSA *			
					(Q9NQV6-4)			
					Isoform 5			
					1-97: MDSKDESSHV...AYVQQDATAQ → MSAYSVPSTFA			
					984-984: I → IQVSEPTASAPSSA *			
					(Q9NQV6-5)			
					Isoform 6			
					511-514: missing			
					984-984: I → IQVSEPTASAPSSA			
					1132-1147: TTTNGNGSSEVHITKP → AGSKVIQNEF...IVFKRISKRI *			
					(Q9NQV6-6)			
PRDM11 (PFM8)	PR domain-containing protein 11		2		Isoform 1 'canonical' sequence	511	aa 149-264	no
					(Q9NQV5-1)			
					Isoform 2			
					1-34: missing *			
					(Q9NQV5-2)			
*PRDM12* (*PFM9*)	PR domain zinc finger protein 12	nucleus	1		(Q9H4Q4)	367	aa 87-207	no
	(PR domain-containing protein 12)							
*PRDM13* ( *PFM10* )	PR domain zinc finger protein 13	nucleus	1		(Q9H4Q3)	707	aa 1-116	no
	(PR domain-containing protein 13)							
*PRDM14*	PR domain zinc finger protein 14	nucleus	1		(Q9GZV8)	571	aa 253-371	no
	(PR domain-containing protein 14)							
*PRDM15* (*C21orf83*; *ZNF298*)	PR domain zinc finger protein 15	nucleus	1		(P57O71)	1,507	aa 406-529	no
	(PR domain-containing protein 15)(Zinc finger protein 298)							
*PRDM16* (*KIaa 1675*; *MEL1*; *PFM13*)	PR domain zinc finger protein 16	nucleus	4		Isoform 1 'canonical' sequence	1,276	aa 83-215	H3K9me1
					(Q9HAZ2-1)			
					Isoform 2 (MEL1L)			
	(PR domain-containing protein 16)				1233-1251: missing *			
					(Q9HAZ2-2)			
					Isoform 3			
					191-191: Q → QV			
	(Transcription factor MEL1)				868-868: missing *			
					(Q9HAZ2-3)			
				Isoform 4				
				Also known as:				
				MEL1S				
				1-184: missing				
				(Q9HAZ2-4)				
*ZNF408* (*PFM14*; *PRDM17*)	Zinc finger protein 408	nucleus			(Q9H9D4)	720		
	(PR domain zinc finger protein 17)							
**Mouse**								
***gene name***	***protein name***	***localization***	***molecular forms***	***alternative promoter(UNIPROT entry)***	***splicing variants(UNIPROT entry)***	***length (aa)***	***PR domain***	***HMT activity***
*Prdm1* (*Blimp1*)	PR domain zinc finger protein 1(B lymphocyte-induced maturation protein 1-Blimp1)	nucleuscytoplasm	5	Isoform 1 'canonical' sequence(Q60636-1)	Isoform 2	856	aa 118-237	no
					Also known as: 1A			
					1-47: MREAYLRCWIFSWKNVWVRP-CQRLHFKTVLLQGSLLYTALDSYSTVQ → MLDLLLEKRVGTTL			
					(Q60636-2)			
				Isoform 3 Also known as: 1B 1-67: missing (Q60636-3)	Isoform 4 (1C)			
					1-47: MREAYLRCWI...TALDSYSTVQ → MTPGVPGHRTQQRPQHISALSDK-AKDCSK			
					(Q60636-4)			
					Isoform 5			
					Also known as: delta exon 7;			
	(Beta-interferon gene positive regulatory domain I-binding factor)(PR domain-containing protein 1)							
					624-666: missing			
					(Q60636-5)			
*Prdm2* (KMT; *Riz1* ; *Znfpr1c1* )	Prdm2 protein	nucleus	1			1,670	aa 34-144	H3K9
*PRDM3* / *Mecom* ( *Evi1* )	MDS1 and EVI1 complex locus protein EVI1 (Ecotropic virus integration site 1 protein-EVI-1)	nucleus	2	Isoform 1 'canonical' sequence		1,042	aa 81-196	H3K9me1
				(P14404-1)				
				Isoform 2				
				(Q9Z1L8-1)				
*Prdm4*	PR domain zinc finger protein 4	nucleus	1		(Q80V63)	803	aa 415-536	no
	(PR domain-containing protein 4)							
*Prdm5*	PR domain zinc finger protein 5	nucleus	1		(Q9CXE0)	599	aa 8-128	no
	(PR domain-containing protein 5)							
*Prdm6* ( *Gm92* ; *Prism* )	Putative histone-lysine *N* -methyltransferase PRDM6 (PR domain zinc finger protein 6) (PR domain-containing protein 6)				Isoform 1 'canonical' sequence	596	aa 248-370	H4K20
					(Q3UZD5-1)			
					Isoform 2			
					1-201: missing			
					(Q3UZD5-2)			
					Isoform 3			
					1-392: missing			
					(Q3UZD5-3)			
					Isoform 4			
					28-58: missing			
					(Q3UZD5-4)			
*Prdm8*	PR domain zinc finger protein 8	nucleus			(Q8BZ97)	687	aa 8-135	H3K9
	(PR domain-containing protein 8)							
*Prdm9* ( *Hst1* ; *Meisetz* )	Histone-lysine *N* -methyltransferase PRDM9	nucleus	4		Isoform 1 'canonical' (Meisetz)	843	aa 246-362	H3K4me3
					(Q96EQ9-1)			
					Isoform 2 (Meisetz-S1)			
	(Hybrid sterility protein 1)				382-404: ELRTEIHPCLLCSLAFSSQKFLT → GGHYYDSLKKKEKREFSLRIFIF			
	(Meiosis-induced factor containing a PR/SET domain and zinc-finger motif)				405-843: missing			
					(Q96EQ9-2)			
					Isoform 3 (Meisetz-S2)			
					382-418: ELRTEIHPCLLCSLAFSSQKFL-TQHMEWNHRTEIFPG → DLFIIICKYT-VAVFRHTRRGSQILLRMVVSHHVVAGI			
	(PR domain zinc finger protein 9)							
					419-843: missing			
	(PR domain-containing protein 9)							
					(Q96EQ9-3)			
					Isoform 4			
					1-121: missing			
					382-404: ELRTEIHPCLLCSLAFSSQKFLT → GGHYYDSLKKKEKREFSLRIFIF			
					405-843: missing			
					(Q96EQ9-4)			
*Prdm10* ( *Gm1112* , *Tris* )	PR domain zinc finger protein 10	nucleus	2		Isoform 1 'canonical' sequence	1,184	aa 200-324	no
					(Q3UTQ7-1)			
					Isoform 2			
					318-341: WYaaSYAEFVNQKIHDISEEE-RKV → QNWIHSCLPARVMIRALSY-KRILP			
	(PR domain-containing protein 10)							
					342-1184: missing			
	(Tristanin)							
					(Q3UTQ7-2)			
*Prdm11*	PR domain-containing protein 11	nucleus	1		(A2AGX3)	565	aa 115-230	no
*Prdm12* (*Gm998*)	PR domain zinc finger protein 12	nucleus	1		(A2AJ77)	365	aa 87-207	no
	(PR domain-containing protein 12)							
*Prdm13*	PR domain zinc finger protein 13	nucleus	2		Isoform 1 'canonical' sequence;	754	aa 5-164	no
					(E9PZZ1-1)			
					Isoform 2			
	(PR domain-containing protein 13)							
					1-48: missing			
					(E9PZZ1-2)			
*Prdm14*	PR domain zinc finger protein 14	nucleus	1		(E9Q3T6)	561	aa 243-360	no
	(PR domain-containing protein 14)							
*PRDM15* (*C21orf83*; *E130018M06Rik*; *ORF62*; *Zfp298*)	PR domain containing 15	nucleus	1			1,174	aa 76-191	no
*PRDM16* (*Kiaa*; 1675; *Mel1*)	PR domain zinc finger protein 16	nucleus	3		Isoform 1 'canonical' sequence	1,275	aa 83-215	H3K9me1
					(A2A935-1)			
					Isoform 2			
					129-129: E → EQ			
	(PR domain-containing protein 16)							
					868-868: Y → YS			
					1174-1176: CVE → HMQ			
					1177-1275: missing *			
	(Transcription factor MEL1)				(A2A935-2)			
					Isoform 3			
					868-868: Y → YS *			
					(A2A935-3)			

* No experimental confirmation available.

## 2. PRDM Proteins in Signal Transduction and Transcription Control

PRDM protein are involved in the transduction of many signals that are responsible for proliferation and differentiation control. PRDM proteins, through the formation of chromatin remodeling complexes, regulate gene expression acting generally as transcription repressors [21,22,23,24]. Some members of the PRDM family show an intrinsic methyltransferase activity [8,25] while others act indirectly, recruiting chromatin remodeling enzymes [22,26,27].

### 2.1. Nuclear Receptor Superfamily Signal Transduction

Nuclear receptors act as ligand-dependent transcription factors, modulating gene expression by direct interaction with well conserved consensus sequences of target genes: *cis*-acting hormone-regulatory elements [28].

**Figure 1:**
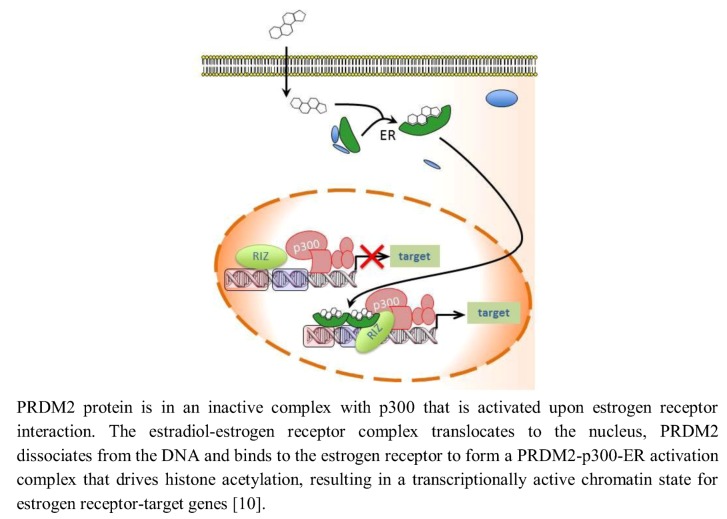
PRDM2 is an estrogen receptor co-activator.

Several findings suggest that the *PRDM2* gene product PRDM2a/RIZ1 is a downstream effector of estrogen action and is related to estrogen-regulated cell proliferation in classical estrogen target tissues. PRDM2 proteins interact with estrogen receptor (ER) through a LXXLL motif and their interaction is dependent on 17β-estradiol treatment [29,30,31]. PRDM2a has *in vitro* histone H3K9 methyltransferase activity and is a weak activator or a repressor of transcription [25,32,33]. It acts as co-activator of estrogen-dependent gene transcription when its methyltransferase activity is inhibited by estradiol (Figure 1) [30,34]. Medici *et al.* in fact demonstrated that PRDM2a is able to bestow estrogen inducibility to a promoter containing an incomplete ERE and a G/C TTGGC motif [29].

Garcia Bassets *et al.* have shown the fundamental role of histone methyltransferase (HMT), including PRDM2a, in maintaining in off-state the promoters regulated by nuclear receptors, such as ERα or androgen receptor (AR). However, the H3-K9 methylation-mediated down-regulation allows the action of lysine-specific demethylase 1 (LSD1) molecules recruited by steroid nuclear receptors ligand, complexed to the same genes [35,36]. Based on this, the opening of regulated genes could involve two crucial events leading to the enhancer effect of the nuclear receptor to promote the DNA unwinding in transcription: the recruitment of topoisomerase β [37] and of OGG1 (8-oxoguanine DNA glycosylase), due to the oxygen radicals produced by the LSD1 action in the removal of the methyl group of dimethyl H3K9 with production of monomethyl H3K9 [38]. This would explain why the PRDM2 (PR-*minus*) form is unable to produce enhancer effects in presence of estradiol, as observed with ERE-Luc reporter assay experiments *in vitro* [39], despite the presence of domains for the recruitment of p300 and p160 co-activators [34]. It might be expected that its full function as co-activator would be due to the presence of the PR domain. In this way, PRDM2a would provide the substratum to histone demethylase near the ERE sequences, thereby supporting and stabilizing the binding of the receptor to DNA, for its ability to recognize flanking sequences and to interact with the AF-2 core sequence in the ER hormone binding domain [29,30].

Moreover, *PRDM2* gene products are endowed with DNA-binding as well as transcription factor-binding activities. In fact PRDM2 was independently isolated as a retinoblastoma-binding protein (RIZ) [40], a DNA-binding protein (MTB-Zf), or as a GATA3 transcription factor binding protein (G3B) [41]. MTB-Zf (essentially identical to PRDM2b) binds to the MTE DNA element GTCATATGAC of human hemeoxygenase-1 gene and can weakly activate transcription [32]. G3B (PRDM2) interacts with the transcription factor GATA-3, regulating the expression of several genes critical for T-cell function and development [42].

Nuclear receptor ligands modulate expression of several *PRDM* genes. In breast cancer cells (MCF-7 cell line), 17β-estradiol stimulation specifically modulates expression of *PRDM2* gene products (PRDM2a and PRDM2b), inducing a shift in the balance of their intracellular concentrations; in particular 17β-estradiol induced a selective decrease in PRDM2a transcript and an increase in total PRDM2 mRNA, accounted by an increase in the PRDM2b form [31]. In fact it was recently demonstrated that the promoter 2 of the *PRDM2* gene contains an estrogen responsive element (ERE) endowed with enhancer activity that is recognized by ERα [43]. Moreover, with the innovative DNA-picked chromatin (DPC) assay, it was possible to observe that estradiol treatment induces a preferential interaction between hormone-responsive *PRDM2* promoter (promoter 2) and the polyadenylation site. Formation of loops has been implicated not only in bringing together far upstream or downstream regions with regulatory or transcribed gene regions, but also in establishing contacts between the 5' and 3' ends of genes, [44,45], in agreement with the now prevalent hypothesis that 3' end-processing factors interact with components of the transcriptional machinery [46]. In the DPC assay, estradiol treatment increased by 60–70% the amount of molecules from exons 9a and 10 (where alternative polyA addition occurs) specifically associated to the captured estradiol-sensitive PRDM2 promoter 2, whereas the recovery of those captured by the estradiol-insensitive PRDM2 promoter (promoter 1) was decreased. The 17β-estradiol remodels the chromatin architecture of *PRDM2* gene locus to create a loop for the mRNA transcription with poliA-exon 9a, leading to the production of oncogenic variants [47]. In non-primary target tissues, however, 17β-estradiol could have an opposite effect, inducing a shift in the PRDM2a/PRDM2b molar ratio in favor of PRDM2a. In fact, in other cell types the hormone stimulation did not affect PRDM2a expression, as in the EPN (epithelial cell line derived from normal human prostate) cell line, or increased it, as SAOS2 (osteosarcoma) cells; serum treatment produced the same effect [48,49].

PRDM2 proteins might also be mediators of androgen effects. In EPN cells, 5α-dihydrotestosterone (DHT) induced a slight increase in cell growth, related to a sharp increase of PRDM2a mRNA and protein concentration. Further investigation could confirm whether *PRDM2* is an androgen responsive gene, because there is an androgen responsive element (ARE) at -361 bp, in the upstream regulatory region of the promoter 1 of *PRDM2* gene [49,50].

PRDM2 proteins might also be active for retinoid action. In fact, in a human promyelocytic leukemia cell line (HL60) treatment with retinoic acid induced a selective expression of PRDM2a and a redistribution of the protein within the nucleus, correlated to the granulocytic differentiation. In HL60 cells, PRDM2a expression was also induced by activation of a retinoid receptor-independent maturation pathway based on retinoid X receptor agonist and protein kinase A synergism [51].

Similarly to PRDM2 acting as co-activator of ERα, PRDM16 stimulates adipogenesis by binding and co-activating, in a ligand-dependent manner, the peroxisome-proliferator-activated receptor γ (PPAR-γ) [52]. PRDM16 also is able to stimulate the function of PGC-1 (Peroxisome proliferator-activated receptor-γ co-activator) α and β in the brown-white fat switch. PRDM16 probably also has a transcriptional repressor activity because the fusion proteins PRDM16/MEL1 or PRDM16/MEL1S-GAL4 DNA-binding domain negatively regulates transcription [7].

### 2.2. Luteinizing Hormone (LH) Signaling

LH stimulates testosterone synthesis in Leydig cells inducing the expression of cytochrome P450 enzymes, 3β-hydroxysteroid dehydrogenase and LH receptor. Prdm8 is a transcriptional repressor that specifically methylates lysine 9 of histone H3. The overexpression of Prdm8 wild-type protein or its mutant deletion, lacking the PR domain, induced a reduction in the expression levels of the steroidogenic enzyme gene *p450c17c* coding for a component of cytochrome P450 family, and of *Luteinizing Hormone Receptor* gene when steroidogenesis was induced in mouse Leydig cells (TM3 cell line) by LH treatment [53]. This evidence suggests that Prdm8 could negatively control steroidogenesis.

### 2.3. Insulin-Like Growth Factor-1 (IGF-1) Signaling

PRDM2a acts as a repressor of a subgroup of genes involved in IGF-1 signaling. A chromatin immunoprecipitation (ChIP) assay showed that PRDM2a down-regulates *IGF-1* expression through a direct binding to its promoter, increasing histone H3K9 methylation. PRDM2a also positively controls insulin-like growth factor-binding protein 2 (*IGFBP-2*) and *SPARC* expression [54]. Moreover, PRDM2a is involved in IGF-1R activation and signal transduction. In fact, forced *PRDM2a* expression in chronic myelogenous leukemia-blast crisis (CML-BC) cell lines decreases activation of IGF-1 receptor and of the downstream signaling components ERK 1/2 and AKT.

### 2.4. NGF Signaling

**Figure 2:**
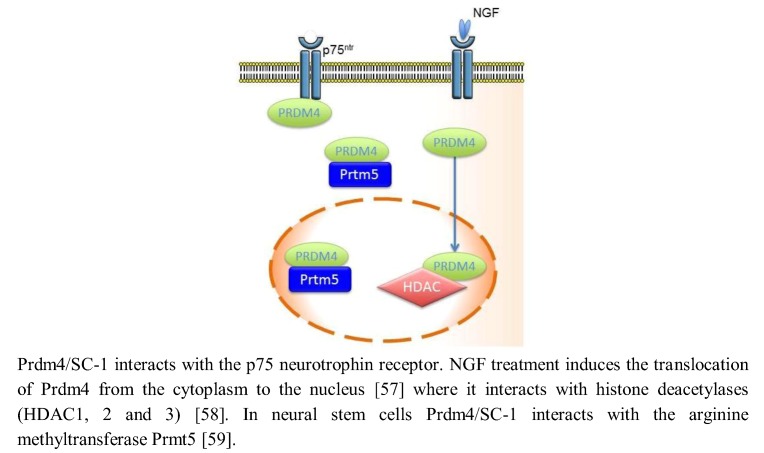
Prdm4/SC-1 (Schwann Cell factor 1) provides a downstream transducer for the effects of nerve growth factor (NGF) through the p75 neurotrophin receptor and forms an epigenetic regulatory complex with Prmt5 that probably maintains the “stem-like” cellular state of a NSC.

Neurotrophins influence a wide number of functions in the nervous system, including neuronal cell survival, cell differentiation and apoptosis, synaptic plasticity, control of axonal guidance and dendrite growth [55,56]. These actions are mediated by neurotrophin binding to two separate receptor classes, the Trk family of tyrosine kinase receptors and the p75 neurotrophin receptor, a member of the tumor necrosis factor receptor superfamily. SC-1 (Schwann Cell factor 1), the *Prdm4* gene product binds to the p75 neurotrophin receptor and provides a downstream transducer for the effects of nerve growth factor (NGF) through this receptor. In fact, NGF treatment of the monkey kidney fibroblast-like cell line (COS) induces a translocation of Prdm4/SC-1 from the cytoplasm to the nucleus that is related to a reduction in bromodeoxyuridine (BrdU) incorporation. The translocation of Prdm4/SC-1 to the nucleus was specific for p75, as NGF binding to the TrkA receptor prevented the nuclear localization of Prdm4/SC-1 (Figure 2) [57]. On the contrary, both TrkA and p75NTR are able to enhance the repressive transcriptional activity of Prdm4/SC-1, implying the role of Prdm4/SC1 as a transducer of NGF signaling by these two receptors [58]. Prdm4/SC-1 acts as a transcriptional repressor forming complexes with trichostatin A (TSA)-sensitive histone deacetylases HDAC1, 2 and 3 and negatively controls cell cycle progression down-regulating cyclin E expression, essential for the G1-S phase transition [58]. In mice cortical neural stem cells (NSCs), Prdm4/SC-1 recruit the chromatin modifier Prmt5 via its *N*-terminus and partly via the PR/SET domain, probably as part of an epigenetic regulatory complex that maintains the “stem-like” cell state of the NSCs by preserving their proliferative capacity and modulating their cell cycle progression [59].

### 2.5. Wnt/β-Catenin and BMP/SMAD Signaling

Wnt signaling is involved in many aspects of embryonic development, such as morphogenetic movements, cell type specification, and patterning. In addition, Wnt/β-catenin regulates pluripotency and differentiation in various stem cell systems, including Embryonic Stem (ES) cells [60]. In murine ES cells, derived from inner cell mass of blastocyst prior the formation of epiblast, the activation of Wnt/β-catenin signaling, through the inhibition of glycogen synthase kinase-3β (GSK3β), leads to the maintaining of pluripotency, induced by bone morphogenetic protein (BMP) or fibroblast growth factor (FGF) via PI3K⁄AKT activation [61,62,63].

PRDM14 is essential for the maintenance of the pluripotent state of human and, potentially, murine ESC, but not for the murine epiSCs (derived from post-implantation epiblast cells), and enhances epigenetic reprogramming of human and murine somatic cells to induced pluripotent stem cells (iPSC) [64].

Co-expression of PRDM1 and PRDM14 is obligatory for the establishment of germ cell lineage [65]. In mammals, the PGCs, the first germ lineage cells are specified in the proximal epiblast [66] and their normal proliferation is ensured by the GSK-3-mediated suppression of Wnt/β-catenin signaling. In PGCs, activation of the Wnt/β-catenin signaling is involved in nuclear reprogramming in culture and nevertheless its aberrant activation leads to germ cell deficiency due to the delay of the cell cycle progression [67]. Wnt signaling alone however, is not sufficient for PGC formation in the absence of BMP. Wnt3, expressed in the epiblast at around E5.5 [68], is a key factor in conferring Bmp4 responsiveness to the epiblasts, giving them the competence to form PGC-like cells. Therefore, Wnt signaling facilitates the response of the epiblast to BMP but itself is not sufficient to induce the PGCs [69]. In the proximal epiblast, BMP/Smad signals induce PRDM1 [70], essential for specification of PGCs [71,72]. PRDM1 complexed with arginine methyltransferase Prtm5, regulates epigenetic reprogramming in germ cell lineages, resulting in high levels of H2A/H4 R3 methylation [26]. Prmt5, a class II arginine methyltransferase, is responsible for the monomethylation of arginine (Rme1) [73] and it has been shown that it methylates cytoplasmic R3 of H2A rather than H4, and that it might be involved in the repression of differentiation genes [74].

Other epigenetic changes, associated with PRDM1 expression, allow PGC to escape the somatic pathway: PGCs show low levels of DNA methylation and H3K9me2 histone marks while acquiring high levels of H3K27me3 modifications [75]. The expression of somatic genes, as Hoxa1 and Hoxb1, is repressed [76] at the same time as the expression of pluripotent marks (Sox2, Pousf1 and Nanog) is re-activated [77].

*In vitro*, the ES cells are capable of differentiating in germ cells [78] and these are at least equivalent to the PGCs that migrate into the fetal gonad and have the potential to undergo meiosis and produce sperm [79]. In embryonic cells fated to become PGCs, PRDM14 is co-expressed with PRDM1 and is critical to the reacquisition of potential pluripotency and successful epigenetic reprogramming [80]. In these cells, PRDM14 expression is regulated by BMP and SMAD signaling and is involved in the establishment of germ cell lineage. The loss of PRDM14 causes defects in genome-wide epigenetic reprogramming with a shift of H3K9me2/H3K27me3 ratio caused by increased expression of the G9a-Like Protein 1 (GLP1, Euchromatic Histone N-Methyltransferase 1, and failure to upregulate Sox2 expression [81]. PRDM1 is therefore not required for the derivation or the maintenance of murine ESCs while it is obligatory for PGC specification and is critical for the maintenance of unipotent germ cells [82].

**Figure 3:**
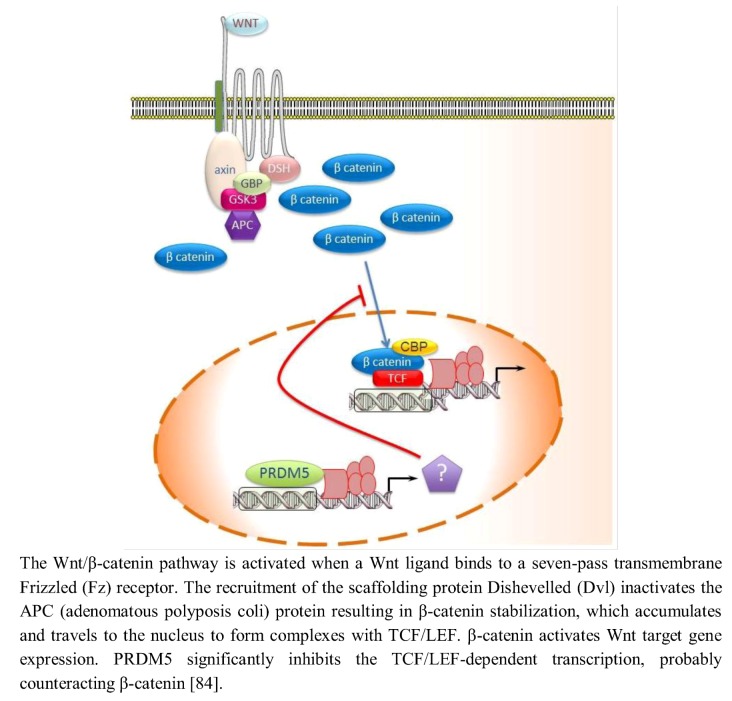
PRDM5 regulates the Wnt/β-catenin signaling in normal cells and in cancer cells.

Aberrant activation of Wnt/β-catenin signaling is also frequently involved in cancers, accompanied with elevated levels of active β-catenin. In addition to genetic defects, epigenetic silencing of Wnt/β-catenin antagonists also leads to aberrant Wnt/β-catenin signaling in tumors [83]. PRDM5 antagonizes the Wnt/β-catenin signaling in normal cells and in cancer cells. By TOPFlash luciferase reporter assay, it was demonstrated that PRDM5 significantly inhibits the T Cell Factor (TCF)/Lymphoid enhancer-binding factor (LEF)-dependent transcription thus hypothesizing that PRDM5 forms a complex with the transcriptional factor TCF (Figure 3) [84]. In agreement with this evidence, the promoter reporter activity of cyclin D1 (*CCND1*), a Wnt/β-catenin downstream target gene whose product binds CDK4, was markedly decreased when PRDM5 was overexpressed [84]. By ChIP assay it was demonstrated that PRDM5 directly binds the promoters of several oncogenes, such as *CDK4* and *TWIST1* and PRDM5 expression resulted in significantly decreased levels of active transcription marks H3K4me3 and acetyl-histone H4 in *CDK4* and *TWIST1* promoters.

### 2.6. Neural Progenitor Maintenance and Differentiation

The nervous system of mammals contains a large number of neurons in a diverse array of neuron classes. Transcription factors play central roles in generating this complexity by controlling neural progenitor cell proliferation, patterning, and defining neuron fate [85,86]. One family that has emerged as important in this regard is the basic helix-loop-helix (bHLH) containing transcription factors [87,88]. For example, evolutionary conserved basic Helix-Loop-Helix (bHLH) transcription factor cascades downstream of Notch signaling is necessary for both the maintenance of neural progenitor cell character and the progression of neurogenesis, while Bhlhb5 olig-related transcription factors Bhlhb5 (also known as Bhlhe22) function predominantly as transcriptional repressors. Bhlhb5 expression is almost exclusively limited to post-mitotic neurons rather than proliferating neural progenitors, hinting at the possibility that Bhlhb5 regulates later aspects of neuronal differentiation [89,90,91].

### 2.7. Notch Signaling

**Figure 4:**
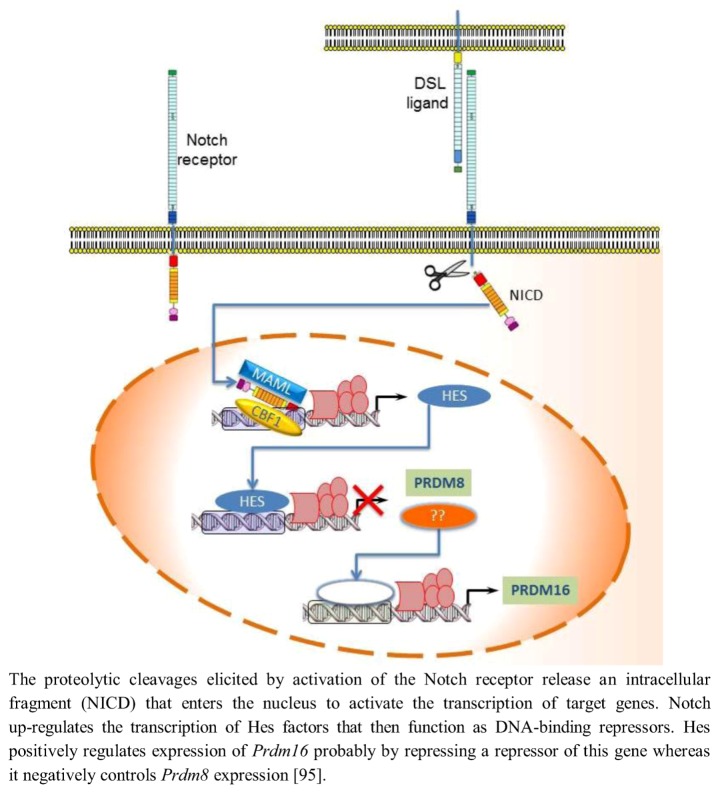
Notch-Hes pathway controls the expression of *Prdm8* and *Prdm16*.

In mammals, Notch activity maintains neural progenitors through an effector pathway consisting of the bHLH Hairy and enhancer of split homologue transcription factors Hes1 and Hes5. Notch up-regulates the transcription of *Hes* factors that then function as DNA-binding repressors and antagonize the expression of *proneural bHLH* genes [92]. Hence, low Notch activity reduces Hes activity and leads to up-regulation of proneural bHLH factors such as Neurogenin2 (Ngn2) and Mammalian achaetescute homolog1 (Mash1); these factors then repress neural progenitor cell maintenance and promote neuronal differentiation [93]. Evidence has revealed an involvement of PRDM protein in the transcriptional regulation mediated by Notch signaling. Hamlet (Ham), the *Drosophila* homolog of mammalian *Prdm3/Evi1* and *Prdm16*, controls olfactory receptor neuron (ORN) development fate by modifying the cellular response to the Notch signals. Ham up-regulating H3K27me3 and down-regulating H3K4me3 directs chromatin-modification events at specific Notch targets, altering the accessibility for Su(H) binding at the enhancer. In nascent ORNs, Ham activity erased the Notch state that was inherited from the parental pNa intermediate precursor cell. This permitted a new and modified response of Notch targets in the subsequent round of Notch signaling [94]. mRNA *in situ* hybridization analysis showed that in the developing murine telencephalon, *Prdm* family genes are expressed at high level in a spatially and temporally restricted manner. The Notch-Hes pathway controls their expression: in particular Hes positively or negatively regulated expression of *Prdm16* and *Prdm8*, respectively. In fact, in Hes-null telencephalon neural differentiation is enhanced, *Prdm8* expression is up-regulated, and *Prdm16* expression is down-regulated. Conversely, electroporation of Hes1 into the developing telencephalon *in utero* up-regulates *Prdm16* expression (Figure 4) implying that *Prdm16* is positively regulated by Hes1 during neurogenesis and expressed in the neural progenitor cell population. As Hes1 protein is believed to act as a transcriptional repressor, positive regulation of Prdm16 by Hes1 may not be direct; it is possible that Hes1 acts by repressing a repressor of Prdm16 expression. Moreover, Prdm16 tags neuronal progenitor cells while Prdm8 does it in the post-mitotic neurons [95].

### 2.8. Neural Circuit Formation

Bhlhb5 binds specific DNA sequence elements and then recruits Prdm8 to inhibit expression of target genes that must be repressed to permit correct development of neural circuits. Mice lacking either *Bhlhb5* or *Prdm8* have strikingly similar cellular and behavioral abnormalities including axonal mistargeting by neurons of the dorsal telencephalon and abnormal itch-like behavior [96], suggesting that Bhlhb5 and Prdm8 are required partners for key aspects of neuronal development. One important target of the Prdm8/Bhlhb5 repressor complex is Cadherin-11 (Cdh11), a cell-cell adhesion molecule involved in neural circuit assembly.

*Prdm8* and *Prdm16* gene products represent therefore, strong new candidates as regulators of neural progenitor cell proliferation and neural differentiation in mammals’ central nervous system (CNS).

### 2.9. TGF-β Signaling

**Figure 5:**
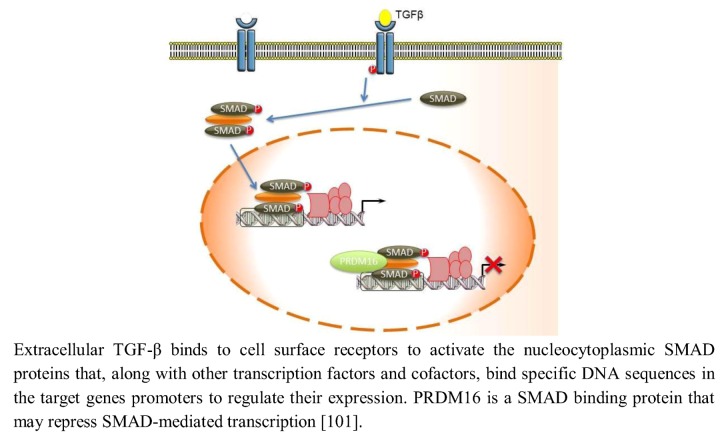
PRDM16 modulates TGF-β signaling.

The Transforming growth factor β (TGF-β) signaling regulates central cell processes, such as proliferation and extracellular matrix production during development of the orofacial region [97,98,99,100]. Extracellular TGF-β binds to cell surface receptors to activate the nucleocytoplasmic SMAD proteins that, along with other transcription factors and cofactors, bind specific DNA sequences in the target genes’ promoters to regulate their expression. PRDM16 is a SMAD-binding protein that can bind a number of different SMADs, including TGF-β and BMP-regulated SMADs, and may modulate their signaling via the TGF-β pathway (Figure 5) [101]. PRDM16 is similar in structure to PRDM3, which has been previously demonstrated to bind and thereby inactivate SMAD3 proteins through its DNA binding domain-1 (Zn-finger domain-1) and repress TGF-β cell growth-inhibitory signaling [102]. PRDM3 and PRDM16, however, bind SMADs and recruit CtBP, which in turn join histone deacetylases (HDACs) to deacetylate histones and repress SMAD mediated transcription [10,21,102,103,104,105].

*Prdm16* is expressed in the murine embryonic secondary palate [101] where it plays a downstream regulatory role in mediating TGF-β signaling, affecting embryonic craniofacial development. Indeed, *Prdm16* knockout murine embryos display a completely penetrant cleft palate [103]. In *Prdm16*^−/−^ fetuses, chromatin immunoprecipitation-promoter microarray analysis (ChIP-Chip) has revealed a gene expression change of markers for bone (Opn) and muscle (Myf-4) development. The expression of Opn, [106], linked to human cases of orofacial clefting, was significantly reduced, while that of Myf-4 was significantly increased, allowing to assume a role for *Prdm16* to myo-, chondro- and/ or osteogenesis in the developing orofacial region, in addition to regulating other processes of normal development. *Prdm16* knockout could cause an abnormal muscle and/or bone development leading to altered morphogenesis of the nascent palatal processes with the failure of reorientation and subsequent separation of the oral and nasal cavities [107].

## 3. PRDM Proteins in the Host Defence

PRDM1 is a transcription repressor that plays a critical role in terminal differentiation of B cells into antibody-secreting plasma cells [11]. PRDM1/Blimp-1 modifies the architecture of chromatin through the interaction with several proteins. The Pro/Ser rich domain interacts with the Groucho family proteins [108], LSD1 (Lysine-Specific Demethylase-1), and the HDAC 2 [24].

Interleukin 21-producing T helper lymphocytes are central to humoral immune response because this cytokine is required for the antibody production induced by IL-6. In B cells, IL-21-treatment induces the expression of signal transducer and activator of transcription 3 (STAT3), required for optimal immunoglobulin production and an up-regulation of PRDM1, the master plasma cell factor [109].

PRDM1 plays also a crucial role in controlling T cell homeostasis [110,111]. In activated T cells, *PRDM1* is induced by IL-2 signaling and inhibits IL-2 production in a negative feedback loop [112]. In naive T helper cells, IL-4 promotes the T_H_2 differentiation and inhibits the T_H_1 differentiation, which induces the down-regulation of IL-2. *PRDM1* is an IL-4 responsive gene that potentiates the IL-2 inhibition. In fact, IL-4-mediated IL-2 suppression was less pronounced in activated, *PRDM1*- deficient T helper cells [113].

Recent studies revealed that the *PRDM1* expression level and, consequently, the secretion of pro-inflammatory cytokines was regulated not only at transcriptional level by activation of T helper cells but also at post-transcriptional level, by enhanced miR-9 expression. The miR-9 is particularly abundant in activated human T helper cells and controls expression of *PRDM1* and B cell lymphoma-6 protein (*Bcl-6*). In fact, suppression of miR-9 led to increased expression of *PRDM1* and *Bcl-6*, which subsequently resulted in diminished secretion of IL-2 and IFN-γ [114].

Another microRNA gene cluster is repressed, in T follicular helper cell (T_FH_ cells), by Bcl-6 to maintain the expression of several T_FH_ genes implicated in lineage commitment [115]. Bcl-6 is a transcriptional repressor that is, at low concentration, recruited by T-bet, a T_H_1-specific T box transcription factor, to maintain the T_H_1 gene-expression profile [116,117]. *PRDM1* is directly targeted by Bcl-6 and is responsible for the repression of a subset of T_FH_ signature gene in T_H_1 cells [118]. Oestreich and colleagues hypothesized a flexibility between T_H_1 and T_FH_-like gene-expression regulated by T-bet-Bcl-6 complex, through the activation or repression of *PRDM1*. In T_H_1 cells, the variations of the ratio between Bcl-6 and T-bet are regulated by the low or high concentration of IL-2. In this way, low concentration of IL-2 enables the Foxo transcription factor to activate *Bcl-6* transcription; Bcl-6 in turn represses the *PRDM1* expression, promoting the expression of T_FH_ signature genes [118].

In addition to controlling the fate of effector T helper cells, PRDM1 cooperates with transcription factor IRF4 for the differentiation of natural T_reg_ cells. Expression of IL-10 is essential for this particular effector function and PRDM1is responsible for the remodeling of active chromatin at the locus *Il10* via trimethylation of histone H3 at Lys27 [119]. In these cells, the *PRDM1* gene is a target for the transcription factor FOXP3, which regulates also the expression of *IRF4* [120,121]. These transcription factors directly regulate *PRDM1* expression in T_reg_ cells by binding two sites in the 3' region and between exons 5 and 6 of *PRDM1* (conserved noncoding sequence 9) [122,123].

*PRDM2* is involved in the regulation of inflammatory response in host defense and might play an important role in inflammatory diseases. In the murine leukemic monocyte macrophage cell line (RAW 267.4) *PRDM2* is a lipopolysaccharide (LPS)-responsive gene that increases the production of TNF-α (Tumor necrosis factor α) and IL-6 by nuclear factor-κB (NF-κB) activation. LPS, in fact, augments the PRDM2a expression via the activation of PI3K/Akt/NF-κB pathway. In turn, PRDM2a increases TNF-α expression level and IL-6 cytokine enhancing NF-κB activation*.* PRDM2a knock-down by RNA interference led, in fact, to the inactivation of NF-κB in response to LPS. TNF-α induced *PRDM2* expression by the activation of NF-κB and AKT signaling. PRDM2a negatively regulates the proliferative activity of TNF-α-treated human monocytic leukemia cells via activation of p53. In fact, PRDM2a forced expression produces an increase of p53 protein expression and silencing of RIZ1 prevented it. On the other hand, a p53 inhibitor enhanced the TNF-α-induced PRDM2a expression [124,125].

*PRDM5* is probably involved with the regulation of hematopoiesis. PRDM5 is in fact able to interact with Growth factor independent 1 (Gfi1) transcription factor, essential for hematopoiesis [126], whose inactivation impaired blood cell formation, causing neutropenia and lymphopenia and release from bone marrow of immature cells [127,128,129]. At molecular level, PRDM5 acts as a sequence-specific DNA binding transcription factor interacting with Gfi1 and recruiting the histone methyltransferase G9a, histone deacetylases HDAC1, 2 and 3 to its target gene promoters [130] to repress transcription.

PRDM5 can also activate some target genes, such as *NOTCH2*, *IL6R*, *MYB* and *c-MYC*, whose transcriptional regulation is also controlled by Gfi1, suggesting that Gfi1-PRDM5 interaction activates rather than represses transcription. Neutropenia-associated PRDM5 sequence variants interfere with its transcriptional activity.

## 4. Box: PRDM Function not Correlated to Signal Transduction

### 4.1. Meiotic Recombination

PRDM9, also referred as Meisetz (Meiosis-induced factor containing PR/SET domain and Zn-finger motif), is a histone methyltransferase acting as a transcription activator of meiosis-specific genes in murine germ cell lineage. PRDM9 has catalytic activity only for trimethylation of lysine 4 of histone H3 and its transactivation activity depends on the methylation activity. The methylation of lysine 4 of histone H3 is a well-characterized feature of transcriptionally active genes [8]. PRDM9 is also involved in meiotic recombination events [131]. Computational analysis revealed that PRDM9 binds with its Zn-finger domain the sequence motifs present in “hotspots” segments of the genome (typically, 2 kb) in which recombination events occur. *Prdm9*-null mice showed arrest of gametes in meiotic prophase I and impaired double-strand break repair [8].

### 4.2. Cytoplasmic Histone Methylation

Histone posttranslational modifications (PTMs) and sequence variants regulate genome function. H3K9 methylation occurs prior to histone incorporation into chromatin. Notably, initial modifications on non-nucleosomal H3 variants can potentiate the action of enzymes as exemplified with SUV39H1 HMT to produce H3K9me3 found in pericentric heterochromatin [132].

It has recently been demonstrated that in mouse embryonic fibroblasts, Prdm3 and Prdm16 are redundant H3K9me1-specific lysine methyltransferase enzymes (KMT) that direct cytoplasmic H3K9me1 methylation. Combined impairment of *Prdm3* and *Prdm16* prevents the nuclear lysine methylation of histone 3 by the SUV39H1 enzyme that reinforce heterochromatin, resulting in disintegration of heterochromatic foci and disruption of the nuclear lamina [133].

Prdm4/SC1 and Prtm5 are located both in the nucleus and cytoplasm of neuroepithelial cells, suggesting that they might act similarly to Prdm3 and Prdm16, inducing methylation of a cytoplasmic pool of newly synthesized histones.

### 4.3. Bone Development

Bone is composed of a highly specialized, mineralized collagenous matrix that provides tensile strength to the skeletal system [134]. *Prdm5* is specifically expressed in the osteoblastic compartment of developing bones and exerts its function along the osteogenic lineage by promoting osteogenic differentiation in culture. Prdm5 targets extracellular matrix (ECM) gene families such as those encoding for collagens and small leucine-rich proteoglycans. Prdm5-bound genes were trimethylated on lysine 9 or 4 of histone 3. The methylation level was higher on lysine 4 than on lysine 9. By association with RNA polymerase II, probably affecting its ability to bind DNA during transcription, Prdm5 sustains the transcription of collagen I genes while the regulation of Decorin expression is mediated by binding to a distal enhancer-like element [135].

### 4.4. Prdm6 Modulates Smooth Muscle Cell (SMC) Phenotype

Prdm6 protein, also named PRISM (*PR* domain in *s*mooth *m*uscle), regulates SMC phenotypic plasticity by suppressing differentiation and maintaining the proliferative potential of vascular SMCs. Prdm6 acts as a transcriptional repressor by interacting with a class I histone deacetylase, heterochromatin protein-1 (HP1-B), a H3K9 specific transcriptional repressor, and the G9a, a ubiquitous H3K9 and K27 methyltransferase, repressing Prdm1-mediated transcription. Prdm6 interacts with transcriptional activators in addition to repressors such as p300, a powerful transcriptional co-activator with intrinsic histone acetyltransferase activity [22].

## 5. Conclusions and Perspectives

*PRDM* gene family has a pivotal role in the control of the proliferation/differentiation switch and expression of its member is relevant during tumorigenesis, when some *PRDM* genes are frequently silenced by genetic or epigenetic mechanisms. Several members of the family express forms containing the SET/PR domain closely involved in cell differentiation and forms without this domain have an oncogenic potential (e.g., *PRDM2, PRDM3* and *PRDM16* gene variants) [13,136]. An imbalance in the amounts of the two products frequently occurs in tumor progression through either disruption or underexpression of the PR-*plus* form or overexpression of the PR-*minus* one*.* Nevertheless, expression of forms missing the PR domain is not only limited to neoplastic transformation and tumor progression. Actually, the significance of the balance between the different forms and the mechanism controlling the ratio is unknown.

PRDM family expanded in vertebrates in parallel with the increased complexity of the genome in higher organisms. *PRDM* genes are grouped in five subfamilies and the genes lying in sister branches of the tree maintain similar gene organization, splicing patterns, and functions. For example, *PRDM2* and *PRDM5*, belonging to the same subfamily (composed of *PRDM2*, *PRDM5*, *PRDM3*, and *PRDM16*), have histone methyltransferase activity and are involved in cell cycle progression regulation [29,30].

By comparing the evolutionary features of *PRDM* genes with their expression in human tissues, it is evident that the newer genes have a lower expression than the older genes and acquire tissue specificity, suggesting a progressive specialization and/or a tighter regulation of their functions. Could the concomitant expression of old and new genes in a tissue suggest a cooperation in the establishment of the phenotype? This behavior is shown by PRDM1 and PRDM14, cooperating during germ cell development, and by PRDM3 and PRDM16, participating to maintain mammalian heterochromatin integrity. We hypothesize that the cooperation is a common characteristic of the *PRDM* gene family. Moreover, we observed (data non published) that *PRDM2* gene siRNA silencing did not produce major phenotypic changes but increased the expression level of other PRDM-family proteins, suggesting that these could have a vicarious role.

PRDM proteins are localized in the nucleus where they participate in the transcriptional regulation of gene expression. However, the function of the PRDM protein in the cytosolic compartment is not completely clarified. Recently it has been demonstrated that PRDM3 and PRDM16 methylate H3K9me1 in the cytosol. Moreover, PRDM2a and Prdm4/SC-1 translocate from the cytosol to the nucleus after retinoic acid and NGF treatment respectively. We hypothesize that other than the role in histone code PRDM proteins targets other cytosolic proteins and control their function. PRDM protein as PRDM2 and PRDM16 are co-activators of the nuclear receptor superfamily and participate in the steroid genomic pathway. No clues are available about the involvement of PRDM proteins in the steroid non genomic pathway.
